# Forecasting the epidemiological trends of COVID-19 prevalence and mortality using the advanced *α*-Sutte Indicator

**DOI:** 10.1017/S095026882000237X

**Published:** 2020-10-05

**Authors:** Yongbin Wang, Chunjie Xu, Sanqiao Yao, Yingzheng Zhao

**Affiliations:** 1 Department of Epidemiology and Health Statistics, School of Public Health, Xinxiang Medical University, Xinxiang, Henan Province, P.R. China; 2 Department of Occupational and Environmental Health, School of Public Health, Capital Medical University, Beijing, P.R. China

**Keywords:** COVID-19

## Abstract

Forecasting the epidemics of the diseases is very valuable in planning and supplying resources effectively. This study aims to estimate the epidemiological trends of the coronavirus disease 2019 (COVID-19) prevalence and mortality using the advanced *α*-Sutte Indicator, and its prediction accuracy level was compared with the most frequently adopted autoregressive integrated moving average (ARIMA) method. Time-series analysis was performed based on the total confirmed cases and deaths of COVID-19 in the world, Brazil, Peru, Canada and Chile between 27 February 2020 and 30 June 2020. By comparing the prediction reliability indices, including the root mean square error, mean absolute error, mean error rate, mean absolute percentage error and root mean square percentage error, the *α*-Sutte Indicator was found to produce lower forecasting error rates than the ARIMA model in all data apart from the prevalence testing set globally. The *α*-Sutte Indicator can be recommended as a useful tool to nowcast and forecast the COVID-19 prevalence and mortality of these regions except for the prevalence around the globe in the near future, which will help policymakers to plan and prepare health resources effectively. Also, the findings of our study may have managerial implications for the outbreak in other countries.

## Introduction

Coronavirus disease 2019 (COVID-19) is an emerging respiratory infectious disease that spreads rapidly from human to human and has presented a pandemic on the global scale [1, 2]. As of 30 June 2020, this disease still continues to take its toll and has led to a major tragedy with 10 185 374 confirmed cases and 503 862 deaths in more than 200 countries, areas or territories [1]. The disease evolves rapidly and has a notable dynamic structure. World Health Organization (WHO) estimated that COVID-19 may still show a rising trend with around 80 000 new notifications per day in the near future [1, 3]. Importantly, the confirmed cases and deaths of this disease vary greatly owing to the differences in disease surveillance and detection capacities among countries, thus causing an obvious underestimation in some countries severely affected by the COVID-19 outbreak [4]. Regrettably, there is a current lack of the determined clinical treatment method and available vaccines for this serious disease [5]. Therefore it is necessary to formulate effective planning for the health infrastructure and services under dynamic demand in order to curb and harness the continued spreading of the COVID-19 pandemic. Accurate forecasting of the epidemiological trends of the COVID-19 prevalence and mortality is essential to manage and instruct the demand to the health system [6–8].

Time-series analysis is of great value in developing hypotheses to understand the past and current epidemic patterns of infectious diseases and to predict the dynamics in the upcoming future [4, 6]. Model-based mathematics and statistics have emerged as useful tools to analyse and estimate time series [9, 10]. For public health officials, such an estimate plays an important role in allocating limited health resources rationally and in directing when and which health interventions should be adopted to alleviate the disease outbreak [4, 6, 11, 12]. Recently, a great number of mathematical and statistical techniques have been deemed as policy-supportive tools to model the prevalence, morbidity and mortality of COVID-19 in different countries [10, 13, 14]. For example, Saba *et al*. used an autoregressive integrated moving average (ARIMA) model and a nonlinear autoregressive artificial neural networks (NARNN) to forecast the prevalence of the COVID-19 outbreak in Egypt [11]. Ceylan *et al*. constructed several suitable ARIMA models to estimate the COVID-19 prevalence in Italy, Spain and France [4]. Chen *et al*. built a Bats-Hosts-Reservoir-People transmission network method to model the potential transmission processes of COVID-19 from bats to humans [7]. Sotgiu *et al*. proposed a new third-degree polynomial curve to simulate the severe acute respiratory syndrome coronavirus 2 (SARS-CoV-2)-related mortality in Italy, Germany, Spain and New York State [8]. Sarkodie *et al*. developed five dynamic statistical techniques to assess the COVID-19 prevalence across China [15]. Cássaro *et al*. constructed a simple approach of growth to model the evolution of the COVID-19 outbreak in various countries [16]. Wan *et al*. used a susceptible exposed infectious recovered (SEIR) method to analyse the epidemic dynamics and trends of COVID-19 in Wuhan [17]. Al-qaness *et al*. developed an optimisation method based on the Adaptive NeuroFuzzy Inference System (ANFIS) to predict the confirmed cases of COVID-19 in China [18]. Bekiros *et al*. built a stereographic Brownian diffusion epidemiology model (SBDiEM) to track the outbreak trends [19]. The epidemiological trends of COVID-19 are driven by many various factors (such as climate change [20], government interventions [21], virus variation [22] etc.), which intimated that the spreading mode of the COVID-19 pandemic is characterised by secular tendencies and irregular fluctuations. For this reason, the above-mentioned methods have a limited ability to consider the tendencies and randomness simultaneously due to their linear or nonlinear assumption, which affects the extrapolation of the results [4].

More recently, a new technical indicator, *α*-Sutte Indicator, is proposed by Ahmar [23], which was originally developed to analyse and estimate the stock movements by taking the opening price, the closing price, the highest price, the lowest price and the volume of transactions on the stock into consideration [23–25]. The *α*-Sutte Indicator may have the potential to accommodate the problems encountered in the above-mentioned models as it has been demonstrated that the *α*-Sutte Indicator can be employed to predict the trends in change of not only the stock movements but also all-time series data [23, 26]. Furthermore, the *α*-Sutte Indicator is relatively easy to evaluate and interpret in that it does not involve complex mathematical or statistical theories. Hence, the decision makers can have an idea of how the forecasting indicator is constructed and can depend more on this predictive tool during the decision-making process. Currently, some countries in the Americas have been hit the hardest with the COVID-19 outbreak and this disease still spreads rapidly, particularly in Brazil, Peru, Canada and Chile [1]. Given the advantage of *α*-Sutte Indicator and the current epidemic status of COVID-19 in the mentioned countries, this study aims to describe the epidemic situation of COVID-19 and to forecast the epidemiological trends of the COVID-19 prevalence and mortality in the above-mentioned countries and worldwide using this advanced *α*-Sutte Indicator. In the meantime, the predictive ability of the *α*-Sutte Indicator was also compared with that of the most common use of ARIMA model in the COVID-19 outbreak forecasting [4, 11, 14, 27–30].

## Materials and methods

### Data collection

The prevalence and mortality time-series data of COVID-19 used in this study corresponded to the period between 27 February 2020 and 30 June 2020, all these data were collected from the Center for Systems Science and Engineering (CSSE) at Johns Hopkins University (https://github.com/CSSEGISandData/COVID-19) and the WHO website (https://www.who.int/emergencies/diseases/novel-coronavirus-2019), and Microsoft Office Excel 2007 was utilised to collate database. Typically, to obtain a robust and effective model in practice, at least 50 observations and preferably 100 observations or more should be used [31]. Consequently, during the model-development process, the data samples from 27 February 2020 to 5 June 2020 (100 observations) were used for the training set, and the remaining 25 samples were taken for the testing set.

The study protocol was approved by the research institutional review board of the Xinxiang Medical University (No: XYLL-2019072), and it was exempt from the institutional review board assessment since all the data analysed in this study were obtained in an anonymous format and any non-essential identifying information were not accessed. In addition, this study meets all the guidelines in the Declaration of Helsinki.

### ARIMA model construction

Time-series prediction aims to use a statistical technique to nowcast and forecast future unknown series by identifying the internal rules between the past and current series. Often, time series displays correlations between successive observations. The ARIMA model is designed to make forecasts by taking correlations existing in the time-series data into consideration [32]. ARIMA model types are listed using the standard notation of ARIMA (p, d, q), herein, p signifies the order of autoregression (AR), d refers to the order of integration and q represents the order of moving average (MA). The ARIMA method is defined for stationary time series. Hence, the stationary conditions of the targeted time series should first be judged by inspecting the time-series plots and by performing the Augmented Dickey–Fuller (ADF) statistic, if the time series displays a trend in change over time and the ADF statistic shows no statistical difference, indicating a non-stationary time series [33]. In this case, the time series requires to be differenced until a stationary series is obtained [34]. Afterward, the values of p and q can roughly be identified by examining the autocorrelation function (ACF) and partial ACF (PACF) graphs of the differenced time series [34]. The ‘Expert Modeler’ in SPSS software and ‘auto.arima()’ in R software have an ability to automatically identify the best-performing ARIMA model by considering the goodness of fit measures such as a larger value of *R*-squared (*R*^2^), a lower value of normalised Bayesian information criterion (NBIC) and the appropriate ACF and PACF graphs of the errors [35, 36]. In this study, both these two tools were used to determine the best model. After that, statistical tests were conducted for the resulting best ARIMA model. The estimated key parameters of the AR and MA should be statistically significant under the *t* test [37, 38]. A Ljung-**Box Q** test was then applied to the residual series produced by the best-fitting ARIMA model, if this statistic provided a *P*-value greater than 0.05, suggesting that the residuals behaved like a white-noise series [37, 38]. At this time, the best ARIMA model passed all the required checking, and then it can be used to implement out-of-sample forecasting. The general forms of the AR process, the MA process and the ARIMA process are given in Eqs. (1), (2), and (3), respectively, below1
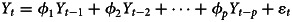
2
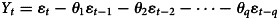
3
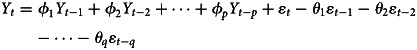
where *ϕ* and *θ* represent the key parameters of AR and MA, respectively, p and q correspond to the orders of AR and MA, respectively,*Y*_*t*_ refers to the observation at time *t* andɛ_*t*_is the residual series that is assumed to be uncorrelated in the final ARIMA model.

### α-Sutte Indicator

*α*-Sutte Indicator is a novel technical analysis based on the Sutte Indicator method that was initially proposed in 2017 by Ahmar [26]. As known, the stock movements failed to be always consistent, sometimes suddenly descended and sometimes suddenly ascended, which caused less accuracy in the domain of stock forecasting using the common technical indicators such as simple moving average (SMA), moving average convergence/divergence (MACD), relative strength index (RSI), stochastic and Bolinger Band [23, 26, 39]. Hence, the development of *α*-Sutte Indicator is expected to overcome the weakness of the mentioned methods in predicting the stock movements by considering five elements of the stock movements, namely price at the time of opening, closing, highest and lowest, along with the volume of transactions [24]. Paralleling the advance in methodology, the *α*-Sutte Indicator is found to be not only limited to forecast the stock movements but can to perform prediction for the time series of data as it can track the dynamic dependence of certain data reasonably well [26, 39, 40]. The formula of *α*-Sutte Indicator can be in the form as below4
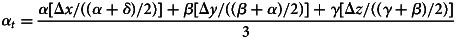
where5
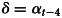
6
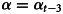
7
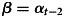
8
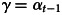
9
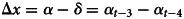
10
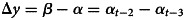
11
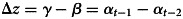
where *α*_*t*_ denotes the observed value at *t* time and *α*_*t*−*k*_ signifies the observed value at (*t*−*k*) time.

### Measuring for the prediction reliability level

The forecasting reliability level between two models was assessed by different measurement metrics, including the scale-dependent measurement metrics (e.g. root mean square error (RMSE) and mean absolute error (MAE)) and the measurement metrics based on percentage errors (e.g. mean error rate (MER), mean absolute percentage error (MAPE) and root mean square percentage error (RMSPE)). A smaller value of these measurement metrics corresponded to the best model and this optimal model was then constructed to produce the out-of-sample forecasting. In the process of predicting data, the 95% confidence limits (CL) of the out-of-sample forecasts were generated by forecasting the in-sample counterparts.12
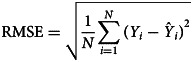
13
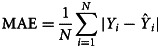
14
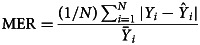
15
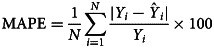
16
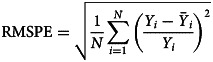
where *Y*_*i*_ refers to the original observations, 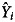 is the prediction values, denotes the average of the original observations and *N* signifies the number of original observations. In this research study, we used SPSS software (version 17.0, IBM Corp, Armonk, NY) and R software (version 4.0.0, R Development Core Team, Vienna, Austria) to construct the ARIMA model and the *α*-Sutte Indicator. The statistical significance level was set at *P* < 0.05.

## Results

### Statistical description

**Fig. 1.**
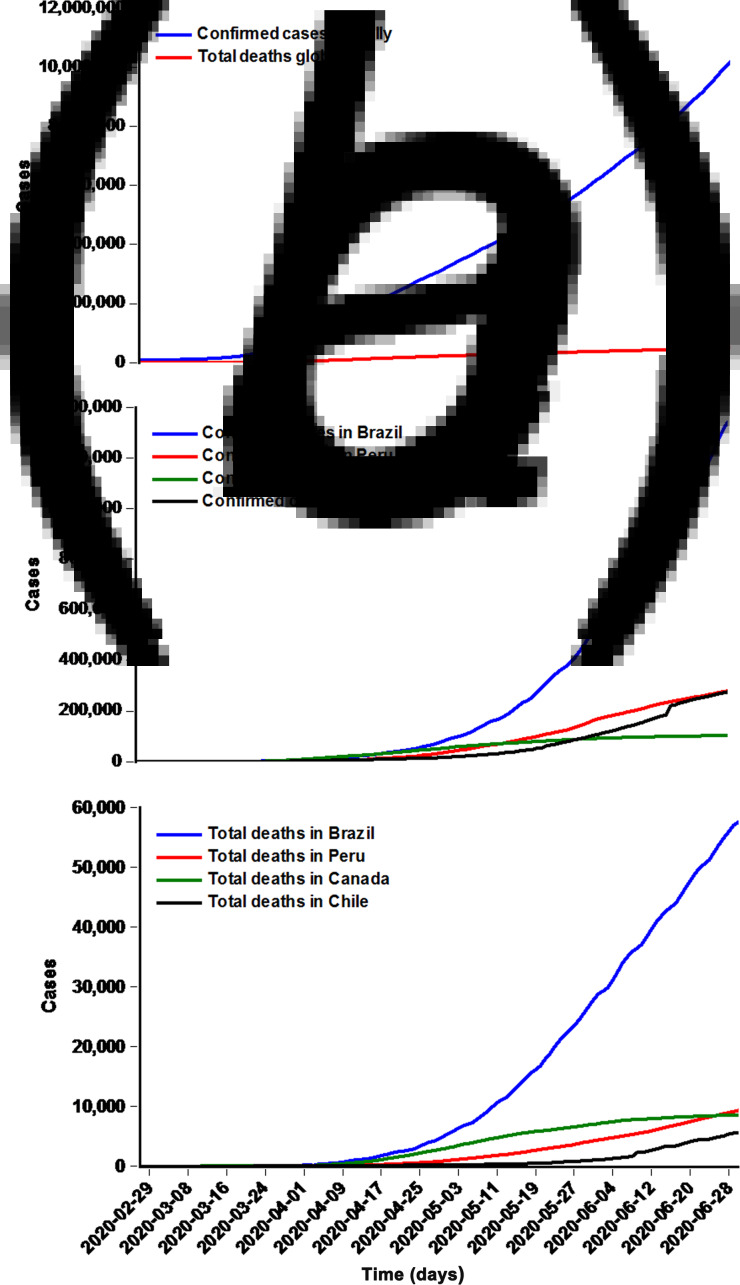
Time series plots displaying the prevalence and mortality cases of COVID-19. (a) The total confirmed cases and deaths worldwide;(b) The total confirmed cases in Brazil, Peru, Canada, and Chile; (c) The total deaths in Brazil, Peru, Canada, and Chile. Worth noting that manycountries, areas or territories recently reconciliated the reported prevalence and mortality data of the COVID-19 outbreak, and thus the prevalenceand mortality data used to build the ARIMA and α-Sutte Indicator models were retrospectively updated on the basis of the additional detailsprovided by WHO, so that we can develop a reliable model for estimating the epidemiological trends of the prevalence and mortality of the COVID-19 outbreak in the upcoming days or weeks.

Between 27 February 2020 and 30 June 2020, the overall confirmed cases were 10 185 374, 1 344 143, 279 419, 103 250 and 275 999 with an average of 81 483, 107 53, 2235, 826 and 2208 case notifications per day in the world, Brazil, Peru, Canada and Chile, respectively (Figs. 1A and 1B). Among them, the reported deaths due to the COVID-19 outbreak have reached 503 862, 57 622, 9317, 8522 and 5575 cases with an average of 4031, 461, 75, 38 and 45 cases per day in the world, Brazil, Peru, Canada and Chile, respectively (Figs. 1A and 1C).

### Building the ARIMA model

**Fig. 2.**
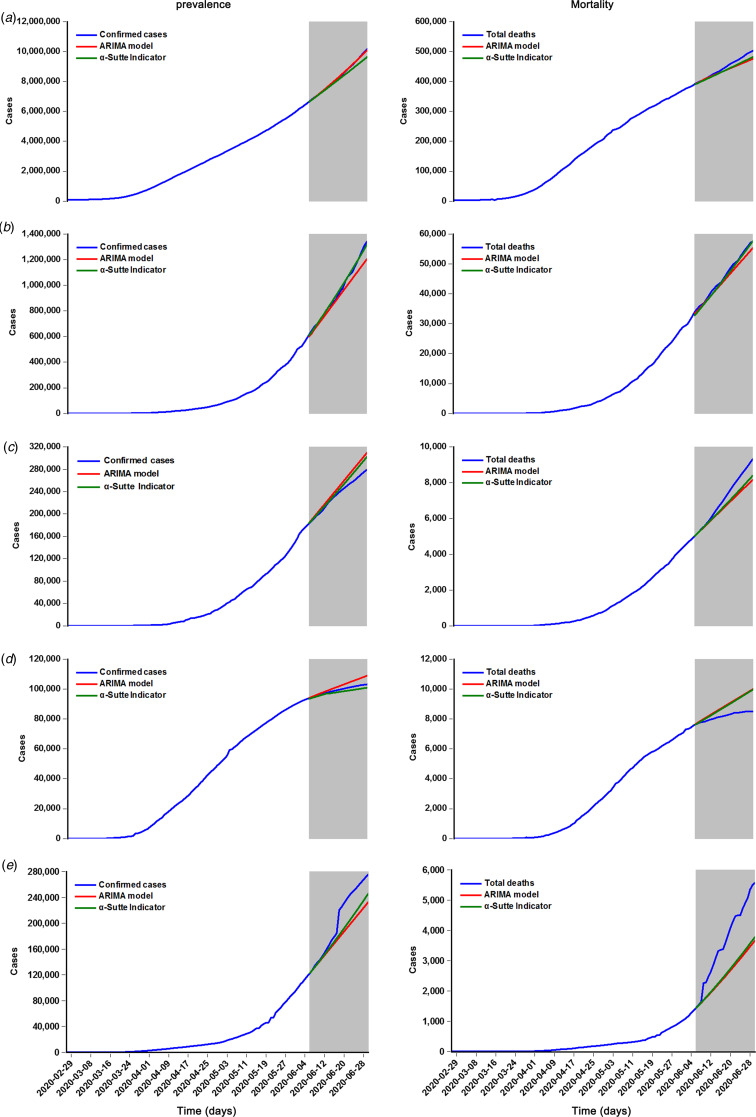
Time series plots displaying the resulting forecasts for the testing sets of COVID-19 prevalence and mortality in the five regions using the α-Sutte Indicator and ARIMA models. (a) The resulting forecasts for the testing sets of the COVID-19 prevalence and mortality globally; (b) The resulting forecasts for the testing sets of the COVID-19 prevalence and mortality in Brazil; (c) The resulting forecasts for the testing sets of the COVID-19 prevalence and mortality in Peru; (d) The resulting forecasts for the testing sets of the COVID-19 prevalence and mortality in Canada; (e) The resulting forecasts for the testing sets of the COVID-19 prevalence and mortality in Chile. Here the forecasts for testing data are plotted as gray shaded area. As seen above, it seemed that the forecasts for the testing sets of both the prevalence and mortality from the α-Sutte Indicator yielded more sufficient prediction accuracy compared with that from the ARIMA model in the five regions except for the result from the testing sets of the COVID-19 prevalence around the globe.

By comparing the best models identified by running the ‘Expert Modeler’ function in SPSS software and auto.ARIMA code in R software, the ARIMA(0,2,(1,7)), ARIMA(0,2,4), ARIMA(0,2,2), ARIMA(1,2,2) and ARIMA(1,2,1) models were considered as the best specifications for forecasting the prevalence time series in the world, Brazil, Peru, Canada and Chile, respectively, as they provided a greater value of the stationary *R*^2^ and *R*^2^, as well as a smaller value of the NBIC in all potential models. Also, as evidenced by the augmented Dickey−Fuller (ADF) test and the ACF and PACF plots for the differenced prevalence series (Supplementary Table S1 and Figs. S1 and S2), it appeared that these selected models are suitable. Further statistical checking suggested that the key parameters of these best models were indicated to be statistically significant under the *t* test (Table 1), and the residuals were indicated to behave like a white-noise series because of a *P*-value greater than 0.05 at different lags under the Box−Ljung Q test and the sample ACF and PACF lying inside the estimated 95% uncertainty limits (Supplementary Table S2 and Fig. S3). These results demonstrated that the chosen best ARIMA models are appropriate and adequate for simulating the prevalence data in these regions. Similarly, we could determine the best-fitting ARIMA models for the mortality time series in these five regions according to the modelling steps, and the resulting results of the identified key parameters and the statistical tests for the best ARIMA models are summarised in Table 2, Supplementary Tables S1 and S3, Figs. S4 and S5. Subsequently, these derived best ARIMA models can be employed to the testing sets to produce out-of-sample projections (Fig. 2).

**Table 1.** The identified best ARIMA models to forecast the epidemiological trend of COVID-19 prevalence in the five regions

Country	Variable	Estimate	s.e.	*t*	*P*	Stationary *R*^2^	*R*^2^	NBIC
Globally	ARIMA(0,2,(1,7)) model							
	MA1	0.777	0.113	6.881	<0.001	0.293	0.987	24.841
	MA7	−0.365	0.130	−2.806	0.006			
Brazil	ARIMA(0,2,(1,2,4)) model							
	MA1	−0.499	0.076	−6.566	<0.001	0.396	1.000	15.312
	MA2	−0.928	0.090	−10.311	<0.001			
	MA4	0.836	0.079	10.582	<0.001			
Peru	ARIMA(1,2,2) model							
	MA1	1.594	0.079	20.096	<0.001	0.757	0.995	16.571
	MA2	−0.681	0.084	−8.089	<0.001			
Canada	ARIMA(1,2,2) model							
	AR1	0.742	0.200	3.710	<0.001	0.449	1.000	11.988
	MA1	1.606	0.169	9.523	<0.001			
	MA2	−0.712	0.123	−5.781	<0.001			
Chile	ARIMA(1,2,2) model							
	AR1	−0.473	0.140	−3.374	0.001			
	MA1	0.857	0.149	5.758	<0.001	0.750	0.999	13.550
	MA2	−0.319	0.138	−2.319	0.023			

ARIMA, autoregressive integrated moving average; AR1, autoregressive at lag one day; MA1, moving average at lag one day; MA2, moving average at lag two days; MA4, moving average at lag four days; MA7, moving average at lag seven days; s.e., standard error; NBIC, normalised Bayesian information criterion.

**Table 2.** The identified best ARIMA models to forecast the epidemiological trend of COVID-19 mortality in the five regions

Country	Variable	Estimate	s.e.	*t*	*P*	Stationary *R*^2^	*R*^2^	NBIC
Globally	ARIMA(0,2,(1,7)) model							
	MA1	0.680	0.178	3.819	<0.001	0.411	1.000	18.141
	MA7	−0.438	0.141	−3.117	0.002			
Brazil	ARIMA(0,2,4) model							
	MA1	0.587	0.073	8.011	<0.001	0.422	1.000	9.687
	MA2	0.482	0.088	5.477	<0.001			
	MA3	0.343	0.090	3.831	<0.001			
	MA4	−0.779	0.075	−10.401	<0.001			
Peru	ARIMA(0,2,2) model							
	MA1	1.696	0.071	24.024	<0.001	0.765	0.986	10.415
	MA2	−0.746	0.072	−10.431	<0.001			
Canada	ARIMA(1,2,2) model							
	AR1	0.705	0.164	4.289	<0.001	0.463	1.000	6.989
	MA1	1.613	0.133	12.145	<0.001			
	MA2	−0.751	0.099	−7.598	<0.001			
Chile	ARIMA(1,2,1) model							
	AR1	−0.633	0.100	−6.361	<0.001	0.625	0.999	4.690
	MA1	0.350	0.125	2.793	0.006			

ARIMA, autoregressive integrated moving average; AR1, autoregressive at lag one day; MA1, moving average at lag one day; MA2, moving average at lag two days; MA3, moving average at lag three days; MA4, moving average at lag four days; MA7, moving average at lag seven days; s.e., standard error; NBIC, normalised Bayesian information criterion.

### Developing the α-Sutte Indicator

Applying the *α*-Sutte Indicator to the prevalence and mortality time series of COVID-19, the resulting forecasts for the testing sets are provided in Figure 2.

### Reliability test between models

To see the forecasting accuracy levels of the *α*-Sutte Indicator method and the ARIMA model, then the comparison of the performance measurement metrics of MAE, MAPE, RMSE, MER and RMSPE from the resulting forecasting results on the testing data using these two models was done. Looking at Table 3, the values of the above-mentioned five indices from the *α*-Sutte Indicator were smaller than the counterparts from the ARIMA models in both the prevalence and mortality testing sets except for that from the prevalence testing set globally, similar results are also illustrated in Figure 2. In the sense that the reliability level in forecasting, the *α*-Sutte Indicator is more appropriate for estimating the epidemiological trends of the COVID-19 prevalence and mortality as compared with the ARIMA model in the study regions with an exception of the prevalence around the globe. Consequently, the next 20-day total confirmed cases and deaths from 1 July 2020 to 20 July 2020 in the study regions were predicted using the *α*-Sutte Indicator on the basis of the data from 27 February 2020 to 30 June 2020 besides the total confirmed cases around the globe, which were done with the ARIMA model (Supplementary Tables S4–S6). The forecasted future 20-day total confirmed cases and deaths may reach 14 153 625 (95% CL 13 293 010 to 15 014 240) and 588 441 (95% CL 587 779 to 589 102) around the globe, respectively, 2 117 890 (95% CL 2 111 135 to 2 124 648) and 74 563 (95% CL 74 365 to 74 761) in Brazil, respectively, 352 946 (95% CL 352 615 to 353 281) and 13 238 (95% CL 13 230 to 13 246) in Peru, respectively, 107 612 (95% CL 107 578 to 107 646) and 8649 (95% CL 8639 to 8659) in Canada, respectively, together with 362 422 (95% CL 357 133 to 367 713) and 8463 (95% CL 8404 to 8532) in Chile, respectively.

**Table 3.** Comparison of accuracy levels measurement of forecasting for the COVID-19 prevalence and mortality between *α*-Sutte indicator and ARIMA methods in the five regions

Country	Model	Accuracy level of forecasting for the prevalence					Accuracy level of forecasting for the mortality				
		MAE	MAPE	RMSE	MER	RMSPE	MAE	MAPE	RMSE	MER	RMSPE
Globally	*α*-Sutte Indicator	176 339.1200	1.9308	237 304.6075	0.0212	0.0249	10 325.7040	2.2179	12 110.7594	0.0231	0.0254
	ARIMA	30 581.6800	0.3588	37 997.2315	0.0037	0.0043	11 335.7600	2.3997	14 549.4620	0.0254	0.0302
	Reduced percentages (%)										
	A *vs*. B	−82.6575	−81.4170	−83.9880	−82.5472	−82.7309	9.7820	8.1969	20.1367	9.9567	18.8976
Brazil	*α*-Sutte Indicator	12 521.7000	1.2969	15 750.682	0.0132	0.0159	709.3080	1.6634	786.0168	0.0155	0.0192
	ARIMA	45 604.9418	4.2679	59 926.2936	0.0481	0.0506	1348.5200	2.7989	1522.5891	0.0295	0.0302
	Reduced percentages (%)										
	A *vs*. B	72.5431	69.6127	73.7166	72.5572	68.5771	47.4010	40.5695	48.3763	47.4576	36.4238
Peru	*α*-Sutte Indicator	7937.308	3.1026	10 762.4731	0.0339	0.0405	416.4634	5.2191	522.6770	0.0587	0.0630
	ARIMA	12 804.0800	5.0966	15 679.8347	0.0547	0.0600	499.0800	6.2362	631.6537	0.0704	0.0758
	Reduced percentages (%)										
	A *vs*. B	38.0095	39.1241	31.3611	38.0256	32.5000	16.5538	16.3096	17.2526	16.6193	16.8865
Canada	*α*-Sutte Indicator	1429.3784	1.4216	1598.0927	0.0144	0.0158	616.0746	7.3669	753.5804	0.0753	0.0894
	ARIMA	2481.6000	2.4564	2998.1281	0.0250	0.0294	666.4400	7.9823	794.5076	0.0815	0.0943
	Reduced percentages (%)										
	A *vs*. B	42.4009	42.1267	46.6970	42.4000	46.2585	7.5574	7.7096	5.1513	7.6074	5.1962
Chile	*α*-Sutte Indicator	20 468.0572	8.6147	26 308.0227	0.1007	0.1080	1064.5131	26.6207	1195.2883	0.2931	0.2843
	ARIMA	25 302.8400	10.6400	32 155.0125	0.1245	0.1309	1116.6400	27.8566	1257.0084	0.3075	0.2975
	Reduced percentages (%)										
	A *vs*. B	19.1077	19.0348	18.1838	19.1165	17.4943	4.6682	4.4367	4.9101	4.6829	4.4370

ARIMA, autoregressive integrated moving average method; MAE, mean absolute error; MAPE, mean absolute percentage error; RMSE, root mean squared error; MER, mean error rate; RMSPE, root mean square percentage error; A denotes the *α*-Sutte Indicator; B represents the ARIMA model.

## Discussion

The eruption of COVID-19 has left extensive and profound impacts around the globe. In view of the current serious challenges raised by the COVID-19 outbreak worldwide, feasible and effective countermeasures are required to prevent and control the rapid increases in the numbers of confirmed cases and deaths. Early nowcasting and forecasting the spreading dynamics of the COVID-19 outbreak are significantly vital for defining strategic choices not only in controlling the transmission of this disease, but also in reducing the outbreak-related deaths, moreover, it benefits for national economic development [4, 8, 41, 42]. As a result, it is necessary to construct the mathematical and statistical models with strong robustness and good reliability to estimate the duration and extent of the COVID-19 outbreak in the most affected countries. Time-series analysis is considerably helpful in forming hypotheses to analyse the epidemiological trends of different diseases and to forecast the epidemic dynamics of the target disease, and subsequently developing a quality control system based on the modelling results [4, 6, 35, 43]. As far as we are aware, this is the only study to perform time-series forecasting for the ongoing trend and extent of the COVID-19 outbreak in the world, Brazil, Peru, Canada and Chile using the advanced *α*-Sutte Indicator, and its predictive performances on the different prevalence and mortality datasets were compared with the ARIMA model which was recommended as the most frequent and powerful tool in the domain of time-series prediction [37, 38]. Our experimental results indicated that *α*-Sutte Indicator has priority over the ARIMA model for forecasting the epidemiological trends of the prevalence and mortality of the COVID-19 outbreak in the mentioned regions except for the epidemiological trends of the COVID-19 prevalence around the globe. Furthermore, the *α*-Sutte Indicator produced a highly accurate prediction as a value less than 10% in all data was presented in the tested forecasting reliability measure of MAPE that is often used to assess the predictive accuracy level [44]. In summary, the satisfactory results from a series of comparative investigations confirm that the *α*-Sutte Indicator has an ability to track the dynamic structures of the prevalence and mortality of the COIVD-19 outbreak, which can help policymakers to determine which and when emergency macroeconomic strategies to be formulated and how to allocate the limited medical and health resources. Meanwhile, this prediction model can also be beneficial in estimating when public health interventions take effects in the study population. For example, the actual prevalence and mortality are beginning to recede, whereas we estimated a higher level using this model, implying that the measures are playing a positive role. Otherwise, additional measures may be required. Besides, given its simple structure, good flexibility and excellent potential to evaluate the data of the *α*-Sutte Indicator, it seems that this method may be transferable to make time-series forecasting for the trends of the epidemiological indicators (such as prevalence, morbidity and mortality) in other countries, territories or areas during the pandemic period or other types of data (such as the data with notable seasonality and periodicity). However, future studies on the additional topic are still required to verify its suitability for the application of the *α*-Sutte Indicator.

The COVID-19 pandemic has been placing an intolerable burden on the health system capacity worldwide [45, 46]. Currently, there is a great concern on whether the countries severely affected by the COVID-19 pandemic have an ability to provide the sufficient number of materials and resources under dynamic demand for the infected people, such as the increased intensive care unit (ICU), the adequate medical supplies, the eventual vaccine and the like [4, 8, 47]. In this study, the upcoming 20-day total cumulative cases and deaths due to the COVID-19 outbreak were estimated using the ARIMA and *α*-Sutte Indicator. The resulting results show that the confirmed cases and deaths may still remain high levels around the globe with a daily average of 181 313 and 4229 cases, respectively, in the next 20 days. Brazil, the second-worst-hit country globally, may still witness an exponential trend with daily 38 687 estimated cases and 847 estimated deaths in the future 20 days. The prior experiences from some countries such as China, Republic of Korea, Italy and Germany have demonstrated that, in the absence of vaccines available, were there no strict control actions such as the lockdown and social distancing measures that have been instituted at the national levels, never would we make the outbreak under control well. Thus, given the current outbreak patterns of COVID-19 in Brazil, the government should continue to implement strict preventive and control strategies, and even more strict interventions, such as continued lockdown, keeping social distancing, an optimisation of the current tools, increasing the numbers of the mobile cabin hospitals, avoiding hospital-related infections, increasing medical personnel, increasing ICU availability, preparing isolation wards, enhancing the awareness of the general public etc. [4, 30, 47–50]. Similar prophylactic measures are also expected to be carried out in Peru and Chile because the daily confirmed cases and deaths have still been noticeably rising in these two countries with daily 3676 and 4321 confirmed cases, coupled with daily 196 and 144 deaths, respectively, and seemingly they required more days to reach the plateau. Contrary to the ongoing trend of the outbreak in the above-mentioned countries, the confirmed cases and deaths are decreasing in Canada with the next 20-day estimates of 218 cases and 6 deaths per day. In all, facing the drastic threats of the COVID-19 pandemic, only under the strict intervention strategies can we hope to tackle such a wide-ranging issue.

## Conclusion

Forecasting the epidemiological trends of the prevalence and mortality of the diseases forms the basis for response to epidemics. In this time-series analysis, we focused on exploring the potential of the advanced *α*-Sutte Indicator and its suitability for the application to the epidemiological trend forecasting of the COVID-19 prevalence and mortality through a series of experiments. Our research suggests that this advanced model can get a more clear perspective of the trends of the epidemiological indicators of the COVID-19 outbreak in the five study regions except for the prevalence data around the globe than the most frequently used ARIMA model. The advanced *α*-Sutte Indicator can be recommended as a useful tool to nowcast and forecast the prevalence and mortality time series of COVID-19, which will be a useful aid for policymakers to plan and prepare health resources effectively, including medical personnel, medical protection facilities, isolation wards and ICU in response to the epidemic patterns of COVID-19 over the upcoming days or weeks. In addition, under the current outbreak trends, feasible and effective strategies are warranted to mitigate the continued spread of COVID-19.

## Data Availability

All the data used in this research can be extracted from the WHO website and the Center for Systems Science and Engineering.

## Supplementary material

For supplementary material accompanying this paper visit http://dx.doi.org/10.1017/S095026882000237X. click here to view supplementary material
