# Supplementary material for: Forecasting the epidemiological trends of COVID-19 prevalence and mortality using the advanced α-Sutte Indicator
Source: Epidemiol Infect. 2020 Oct 5;148:e236. doi: 10.1017/S095026882000237X (PMC7562786; doi:10.1017/S095026882000237X)
Supplement: Supplementary file 1 [file S095026882000237Xsup001.docx]

Supplementary material: Forecasting the epidemiological trends of COVID-19 prevalence and mortality using the advanced α-Sutte Indicator

Yongbin Wang^1^^¶,*^, Chunjie Xu^2¶^, Sanqiao Yao^1^ and Yingzheng Zhao^1^

^1^ Department of Epidemiology and Health Statistics, School of Public Health, Xinxiang Medical University, Xinxiang, Henan Province, P.R. China

^2^ Department of Occupational and Environmental Health, School of Public Health, Capital Medical University, Beijing, P.R. China

^*^ Corresponding author: Yongbin Wang ([wybwho@163.com](mailto:wybwho@163.com)), No. 601 Jinsui road, Hongqi district, Xinxiang city, Henan province, 453003, P.R. China; Tel: +86-373-3831646

^¶^ These authors contributed equally to this paper

**Table S1.** The augmented Dickey–Fuller (ADF) test for the actual series and differenced series in the five regions.

| Degree of difference | Globally | |  | Brazil | |  | Peru | |  | Canada | |  | Chile | |
| --- | --- | --- | --- | --- | --- | --- | --- | --- | --- | --- | --- | --- | --- | --- |
|  | ADF | *p*-value |  | ADF | *p*-value |  | ADF | *p*-value |  | ADF | *p*-value |  | ADF | *p*-value |
| **Prevalence data** | | | | | | | | | | | | | | |
| Actual series | 1.390 | 0.957 |  | 6.211 | 1.000 |  | 8.131 | 1.000 |  | 2.258 | 0.994 |  | 14.047 | 1.000 |
| First-order difference | 1.176 | 0.935 |  | 0.057 | 0.631 |  | -5.533 | <0.001 |  | -1.121 | 0.256 |  | -0.257 | 0.531 |
| Second-order difference | -8.649 | <0.001 |  | -10.124 | <0.001 |  | -15.572 | <0.001 |  | -13.171 | <0.001 |  | -15.779 | <0.001 |
| **Mortality data** | | | | | | | | | | | | | | |
| Actual series | 2.032 | 0.990 |  | 4.384 | 1.000 |  | 4.657 | 1.000 |  | 2.551 | 0.997 |  | 12.771 | 1.000 |
| First-order difference | -0.910 | 0.323 |  | 0.032 | 0.623 |  | -7.990 | <0.001 |  | -0.864 | 0.338 |  | 1.587 | 0.971 |
| Second-order difference | -10.791 | <0.001 |  | -8.868 | <0.001 |  | -15.170 | <0.001 |  | -12.962 | <0.001 |  | -10.983 | <0.001 |

**Table S2.** Box-Ljung Q test for the prevalence residual series from the identified best ARIMA models in the five regions.

| Lags | Globally | |  | Brazil | |  | Peru | |  | Canada | |  | Chile | |
| --- | --- | --- | --- | --- | --- | --- | --- | --- | --- | --- | --- | --- | --- | --- |
|  | Box-Ljung Q | *p* |  | Box-Ljung Q | *p* |  | Box-Ljung Q | *p* |  | Box-Ljung Q | *p* |  | Box-Ljung Q | *p* |
| 1 | 0.003 | 0.960 |  | 0.182 | 0.670 |  | 0.508 | 0.476 |  | 0.109 | 0.741 |  | 0.283 | 0.595 |
| 2 | 1.190 | 0.552 |  | 0.471 | 0.790 |  | 0.863 | 0.650 |  | 0.135 | 0.935 |  | 0.428 | 0.807 |
| 3 | 2.759 | 0.430 |  | 1.591 | 0.662 |  | 0.864 | 0.834 |  | 0.767 | 0.857 |  | 0.430 | 0.934 |
| 4 | 3.222 | 0.521 |  | 3.306 | 0.508 |  | 0.932 | 0.920 |  | 1.070 | 0.899 |  | 0.746 | 0.946 |
| 5 | 3.348 | 0.647 |  | 5.900 | 0.316 |  | 1.217 | 0.943 |  | 1.084 | 0.956 |  | 1.769 | 0.880 |
| 6 | 4.764 | 0.574 |  | 6.100 | 0.412 |  | 1.535 | 0.957 |  | 1.330 | 0.970 |  | 4.033 | 0.672 |
| 7 | 6.821 | 0.448 |  | 6.498 | 0.483 |  | 2.214 | 0.947 |  | 1.360 | 0.987 |  | 4.647 | 0.703 |
| 8 | 6.822 | 0.556 |  | 7.228 | 0.512 |  | 2.601 | 0.957 |  | 1.656 | 0.990 |  | 4.648 | 0.794 |
| 9 | 7.148 | 0.622 |  | 8.076 | 0.527 |  | 2.726 | 0.974 |  | 2.543 | 0.980 |  | 8.494 | 0.485 |
| 10 | 7.550 | 0.673 |  | 8.485 | 0.582 |  | 2.814 | 0.985 |  | 2.573 | 0.990 |  | 9.449 | 0.490 |
| 11 | 9.951 | 0.535 |  | 8.496 | 0.668 |  | 2.887 | 0.992 |  | 4.991 | 0.932 |  | 9.567 | 0.570 |
| 12 | 9.980 | 0.618 |  | 10.268 | 0.592 |  | 2.945 | 0.996 |  | 6.825 | 0.869 |  | 9.652 | 0.646 |

**Table S3.** Box-Ljung Q test for the mortality residual series from the identified best ARIMA models in the five regions.

| Lags | Globally | |  | Brazil | |  | Peru | |  | Canada | |  | Chile | |
| --- | --- | --- | --- | --- | --- | --- | --- | --- | --- | --- | --- | --- | --- | --- |
|  | Box-Ljung Q | *p* |  | Box-Ljung Q | *p* |  | Box-Ljung Q | *p* |  | Box-Ljung Q | *p* |  | Box-Ljung Q | *p* |
| 1 | 2.634 | 0.105 |  | 0.167 | 0.683 |  | 0.000 | 0.992 |  | 0.022 | 0.881 |  | 0.001 | 0.970 |
| 2 | 3.495 | 0.174 |  | 0.205 | 0.903 |  | 0.084 | 0.959 |  | 0.092 | 0.955 |  | 0.065 | 0.968 |
| 3 | 3.526 | 0.317 |  | 0.306 | 0.959 |  | 0.417 | 0.937 |  | 0.203 | 0.977 |  | 0.120 | 0.989 |
| 4 | 6.460 | 0.167 |  | 2.900 | 0.575 |  | 0.966 | 0.915 |  | 0.537 | 0.970 |  | 0.442 | 0.979 |
| 5 | 8.133 | 0.149 |  | 2.933 | 0.710 |  | 1.211 | 0.944 |  | 0.562 | 0.990 |  | 0.519 | 0.991 |
| 6 | 10.542 | 0.104 |  | 2.956 | 0.814 |  | 1.505 | 0.959 |  | 1.283 | 0.973 |  | 13.562 | 0.035 |
| 7 | 11.390 | 0.122 |  | 11.694 | 0.111 |  | 1.622 | 0.978 |  | 1.586 | 0.979 |  | 13.593 | 0.059 |
| 8 | 11.391 | 0.181 |  | 11.796 | 0.161 |  | 1.630 | 0.990 |  | 1.867 | 0.985 |  | 13.677 | 0.091 |
| 9 | 11.399 | 0.249 |  | 12.996 | 0.163 |  | 1.661 | 0.996 |  | 2.166 | 0.989 |  | 13.706 | 0.133 |
| 10 | 11.400 | 0.327 |  | 14.975 | 0.133 |  | 1.667 | 0.998 |  | 3.003 | 0.981 |  | 14.787 | 0.140 |
| 11 | 12.885 | 0.301 |  | 14.990 | 0.183 |  | 1.669 | 0.999 |  | 3.077 | 0.990 |  | 15.354 | 0.167 |
| 12 | 13.210 | 0.354 |  | 14.997 | 0.242 |  | 1.670 | 1.000 |  | 4.622 | 0.969 |  | 18.566 | 0.100 |

**Table S4.** The identified best ARIMA model based on the whole data to forecast the epidemiological trend of COVID-19 prevalence around the globe.

| Country | Variable | Estimate | S.E. | t | *p* | Stationary R^2^ | R^2^ | NBIC |
| --- | --- | --- | --- | --- | --- | --- | --- | --- |
| Globally | ARIMA(0,2,(1,7,14)) model | | | | | | | |
|  | MA1 | 0.520 | 0.119 | 4.368 | <0.001 | 0.431 | 1.000 | 18.589 |
|  | MA7 | -0.392 | 0.086 | -4.541 | <0.001 |  |  |  |
|  | MA14 | -0.457 | 0.111 | -4.102 | <0.001 |  |  |  |

ARIMA Autoregressive integrated moving average, MA1 Moving average at lag one day, MA7 Moving average at lag seven days, MA14 Moving average at lag 14 days, S.E. Standard error, NBIC Normalized Bayesian information criterion.

**Table S5.** Forecasting of the COVID-19 prevalence for the next twenty days in the five regions using the α-Sutte Indicator.

| Time |  | Globally^*^ | | |  | Brazil | | |  | Peru | | |  | Canada | | |  | Chile | | |
| --- | --- | --- | --- | --- | --- | --- | --- | --- | --- | --- | --- | --- | --- | --- | --- | --- | --- | --- | --- | --- |
|  |  | Forecast | 95% LCI | 95% UCI |  | Forecast | 95% LCI | 95% UCI |  | Forecast | 95% LCI | 95% UCI |  | Forecast | 95% LCI | 95% UCI |  | Forecast | 95% LCI | 95% UCI |
| 1/07/20 |  | 10352842 | 10332629 | 10373055 |  | 1383421 | 1376416 | 1390426 |  | 283048 | 282713 | 283384 |  | 103460 | 103426 | 103494 |  | 280245 | 274892 | 285598 |
| 2/07/20 |  | 10545692 | 10509365 | 10582018 |  | 1420068 | 1413066 | 1427069 |  | 286633 | 286297 | 286969 |  | 103682 | 103648 | 103716 |  | 284436 | 279084 | 289789 |
| 3/07/20 |  | 10735999 | 10681773 | 10790225 |  | 1456000 | 1449002 | 1462996 |  | 290203 | 289867 | 290539 |  | 103898 | 103864 | 103932 |  | 288619 | 283267 | 293970 |
| 4/07/20 |  | 10923650 | 10849613 | 10997688 |  | 1493784 | 1486791 | 1500773 |  | 293820 | 293485 | 294156 |  | 104115 | 104081 | 104149 |  | 292857 | 287507 | 298206 |
| 5/07/20 |  | 11127226 | 11031550 | 11222901 |  | 1531042 | 1524056 | 1538024 |  | 297433 | 297098 | 297769 |  | 104334 | 104300 | 104368 |  | 297091 | 291743 | 302439 |
| 6/07/20 |  | 11324996 | 11205982 | 11444010 |  | 1568497 | 1561519 | 1575471 |  | 301056 | 300721 | 301392 |  | 104551 | 104517 | 104585 |  | 301340 | 295994 | 306686 |
| 7/07/20 |  | 11517859 | 11373934 | 11661784 |  | 1606461 | 1599492 | 1613426 |  | 304696 | 304360 | 305031 |  | 104769 | 104735 | 104803 |  | 305611 | 300267 | 310955 |
| 8/07/20 |  | 11710191 | 11535546 | 11884836 |  | 1644475 | 1637516 | 1651430 |  | 308343 | 308008 | 308678 |  | 104987 | 104953 | 105021 |  | 309893 | 304552 | 315234 |
| 9/07/20 |  | 11904979 | 11694808 | 12115149 |  | 1682736 | 1675789 | 1689680 |  | 312001 | 311666 | 312337 |  | 105205 | 105171 | 105239 |  | 314190 | 308852 | 319529 |
| 10/07/20 |  | 12105529 | 11855799 | 12355259 |  | 1721262 | 1714327 | 1728194 |  | 315671 | 315336 | 316007 |  | 105423 | 105389 | 105457 |  | 318503 | 313168 | 323839 |
| 11/07/20 |  | 12310976 | 12018221 | 12603731 |  | 1759969 | 1753048 | 1766888 |  | 319351 | 319016 | 319687 |  | 105642 | 105608 | 105676 |  | 322830 | 317499 | 328162 |
| 12/07/20 |  | 12518211 | 12179389 | 12857034 |  | 1798902 | 1791996 | 1805807 |  | 323042 | 322708 | 323377 |  | 105860 | 105826 | 105894 |  | 327172 | 321844 | 332500 |
| 13/07/20 |  | 12724751 | 12337143 | 13112359 |  | 1838055 | 1831164 | 1844945 |  | 326743 | 326409 | 327079 |  | 106079 | 106045 | 106113 |  | 331528 | 326205 | 336853 |
| 14/07/20 |  | 12928876 | 12490020 | 13367731 |  | 1877412 | 1870538 | 1884286 |  | 330455 | 330122 | 330791 |  | 106298 | 106264 | 106332 |  | 335899 | 330580 | 341220 |
| 15/07/20 |  | 13133001 | 12636374 | 13629627 |  | 1916981 | 1910125 | 1923838 |  | 334178 | 333845 | 334514 |  | 106516 | 106482 | 106550 |  | 340284 | 334969 | 345600 |
| 16/07/20 |  | 13337125 | 12776955 | 13897296 |  | 1956758 | 1949920 | 1963597 |  | 337911 | 337578 | 338247 |  | 106735 | 106701 | 106769 |  | 344684 | 339373 | 349995 |
| 17/07/20 |  | 13541250 | 12912451 | 14170050 |  | 1996738 | 1989920 | 2003558 |  | 341654 | 341322 | 341990 |  | 106954 | 106920 | 106988 |  | 349097 | 343792 | 354404 |
| 18/07/20 |  | 13745375 | 13043399 | 14447351 |  | 2036922 | 2030124 | 2043722 |  | 345408 | 345076 | 345743 |  | 107173 | 107139 | 107207 |  | 353525 | 348225 | 358826 |
| 19/07/20 |  | 13949500 | 13170166 | 14728834 |  | 2077307 | 2070530 | 2084086 |  | 349172 | 348840 | 349507 |  | 107393 | 107359 | 107427 |  | 357966 | 352672 | 363263 |
| 20/07/20 |  | 14153625 | 13293010 | 15014240 |  | 2117890 | 2111135 | 2124648 |  | 352946 | 352615 | 353281 |  | 107612 | 107578 | 107646 |  | 362422 | 357133 | 367713 |

* signifies the forecasts around the globe based on the whole COVID-19 prevalence data using the best ARIMA(0,2,(1,7,14)) model, 95% LCI 95% lower confidence limit, 95% UCI 95% upper confidence limit.

**Table S6.** Forecasting of the COVID-19 mortality for the next twenty days in the five regions using the α-Sutte Indicator.

| Time | Globally | | |  | Brazil | | |  | Peru | | |  | Canada | | |  | Chile | | |
| --- | --- | --- | --- | --- | --- | --- | --- | --- | --- | --- | --- | --- | --- | --- | --- | --- | --- | --- | --- |
|  | Forecast | 95% LCI | 95% UCI |  | Forecast | 95% LCI | 95% UCI |  | Forecast | 95% LCI | 95% UCI |  | Forecast | 95% LCI | 95% UCI |  | Forecast | 95% LCI | 95% UCI |
| 1/07/20 | 508125 | 507461 | 508789 |  | 58513 | 58313 | 58713 |  | 9504 | 9496 | 9512 |  | 8528 | 8518 | 8538 |  | 5747 | 5681 | 5813 |
| 2/07/20 | 512264 | 511600 | 512927 |  | 59371 | 59171 | 59571 |  | 9695 | 9687 | 9703 |  | 8535 | 8525 | 8545 |  | 5882 | 5817 | 5949 |
| 3/07/20 | 516397 | 515733 | 517061 |  | 60143 | 59943 | 60343 |  | 9883 | 9875 | 9891 |  | 8541 | 8531 | 8551 |  | 6008 | 5943 | 6075 |
| 4/07/20 | 520593 | 519929 | 521256 |  | 60989 | 60789 | 61189 |  | 10073 | 10065 | 10081 |  | 8547 | 8537 | 8557 |  | 6154 | 6089 | 6221 |
| 5/07/20 | 524765 | 524101 | 525429 |  | 61820 | 61620 | 62020 |  | 10265 | 10257 | 10273 |  | 8554 | 8544 | 8564 |  | 6292 | 6227 | 6359 |
| 6/07/20 | 528949 | 528285 | 529613 |  | 62642 | 62442 | 62842 |  | 10457 | 10449 | 10465 |  | 8560 | 8550 | 8570 |  | 6430 | 6365 | 6497 |
| 7/07/20 | 533150 | 532486 | 533813 |  | 63481 | 63281 | 63680 |  | 10650 | 10642 | 10658 |  | 8566 | 8556 | 8576 |  | 6572 | 6508 | 6639 |
| 8/07/20 | 537353 | 536689 | 538016 |  | 64317 | 64117 | 64516 |  | 10844 | 10836 | 10852 |  | 8573 | 8563 | 8583 |  | 6712 | 6649 | 6780 |
| 9/07/20 | 541565 | 540901 | 542228 |  | 65155 | 64955 | 65354 |  | 11039 | 11031 | 11047 |  | 8579 | 8569 | 8589 |  | 6854 | 6791 | 6922 |
| 10/07/20 | 545787 | 545123 | 546450 |  | 65998 | 65798 | 66197 |  | 11235 | 11227 | 11243 |  | 8585 | 8575 | 8595 |  | 6997 | 6934 | 7065 |
| 11/07/20 | 550015 | 549352 | 550678 |  | 66842 | 66643 | 67041 |  | 11431 | 11423 | 11439 |  | 8592 | 8582 | 8602 |  | 7140 | 7078 | 7208 |
| 12/07/20 | 554253 | 553589 | 554915 |  | 67689 | 67490 | 67888 |  | 11629 | 11621 | 11637 |  | 8598 | 8588 | 8608 |  | 7284 | 7222 | 7352 |
| 13/07/20 | 558498 | 557835 | 559161 |  | 68539 | 68340 | 68738 |  | 11827 | 11819 | 11835 |  | 8604 | 8594 | 8614 |  | 7429 | 7368 | 7497 |
| 14/07/20 | 562751 | 562088 | 563414 |  | 69392 | 69193 | 69591 |  | 12026 | 12018 | 12034 |  | 8611 | 8601 | 8621 |  | 7575 | 7514 | 7643 |
| 15/07/20 | 567013 | 566350 | 567675 |  | 70247 | 70049 | 70446 |  | 12226 | 12218 | 12234 |  | 8617 | 8607 | 8627 |  | 7721 | 7660 | 7790 |
| 16/07/20 | 571283 | 570620 | 571945 |  | 71105 | 70907 | 71304 |  | 12427 | 12419 | 12435 |  | 8623 | 8613 | 8633 |  | 7868 | 7808 | 7937 |
| 17/07/20 | 575560 | 574897 | 576222 |  | 71966 | 71768 | 72164 |  | 12629 | 12621 | 12636 |  | 8630 | 8620 | 8640 |  | 8016 | 7956 | 8084 |
| 18/07/20 | 579846 | 579183 | 580507 |  | 72829 | 72631 | 73027 |  | 12831 | 12823 | 12839 |  | 8636 | 8626 | 8646 |  | 8164 | 8105 | 8233 |
| 19/07/20 | 584139 | 583477 | 584801 |  | 73695 | 73497 | 73893 |  | 13034 | 13026 | 13042 |  | 8642 | 8632 | 8652 |  | 8313 | 8254 | 8382 |
| 20/07/20 | 588441 | 587779 | 589102 |  | 74563 | 74365 | 74761 |  | 13238 | 13230 | 13246 |  | 8649 | 8639 | 8659 |  | 8463 | 8404 | 8532 |

95% LCI 95% lower confidence limit, 95% UCI 95% Upper confidence limit.


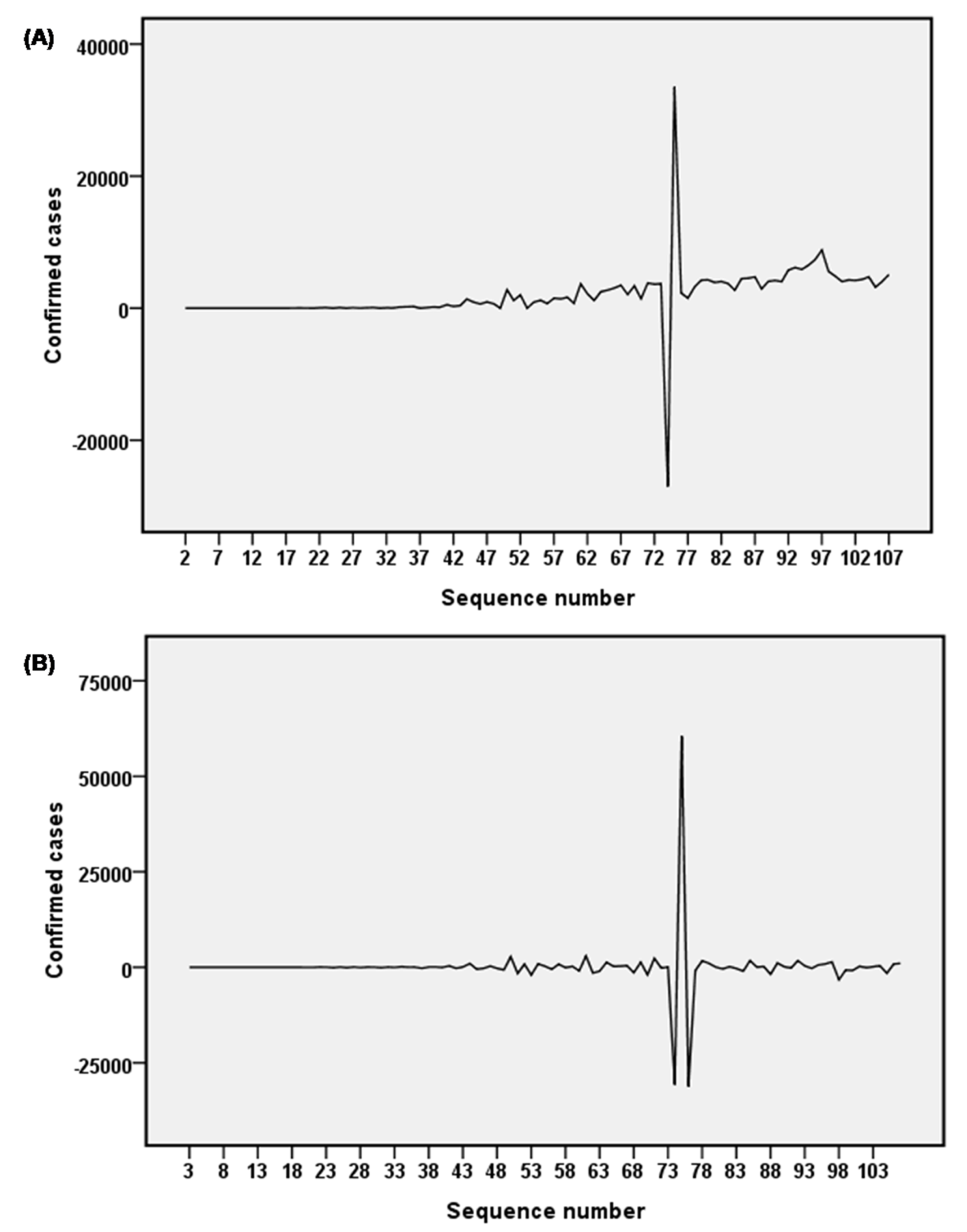


**Fig. S1.** Time series plots showing the differenced confirmed cases in Peru. (A) The first-order differenced series plot; (B) The second-order differenced series plot. It can be seen that the time series the first-order differenced series still displayed a certain trend, so it is required to perform the second-order difference to remove this trend. After differencing twice, the time series plot achieved stationarity. At this time, this stationary sequence could be used to identify the best-fitting ARIMA model for predicting the total confirmed cases in Peru. In an attempt, it seems that the ARIMA(1,2,2) model is adequate for modeling the total confirmed cases in Peru.


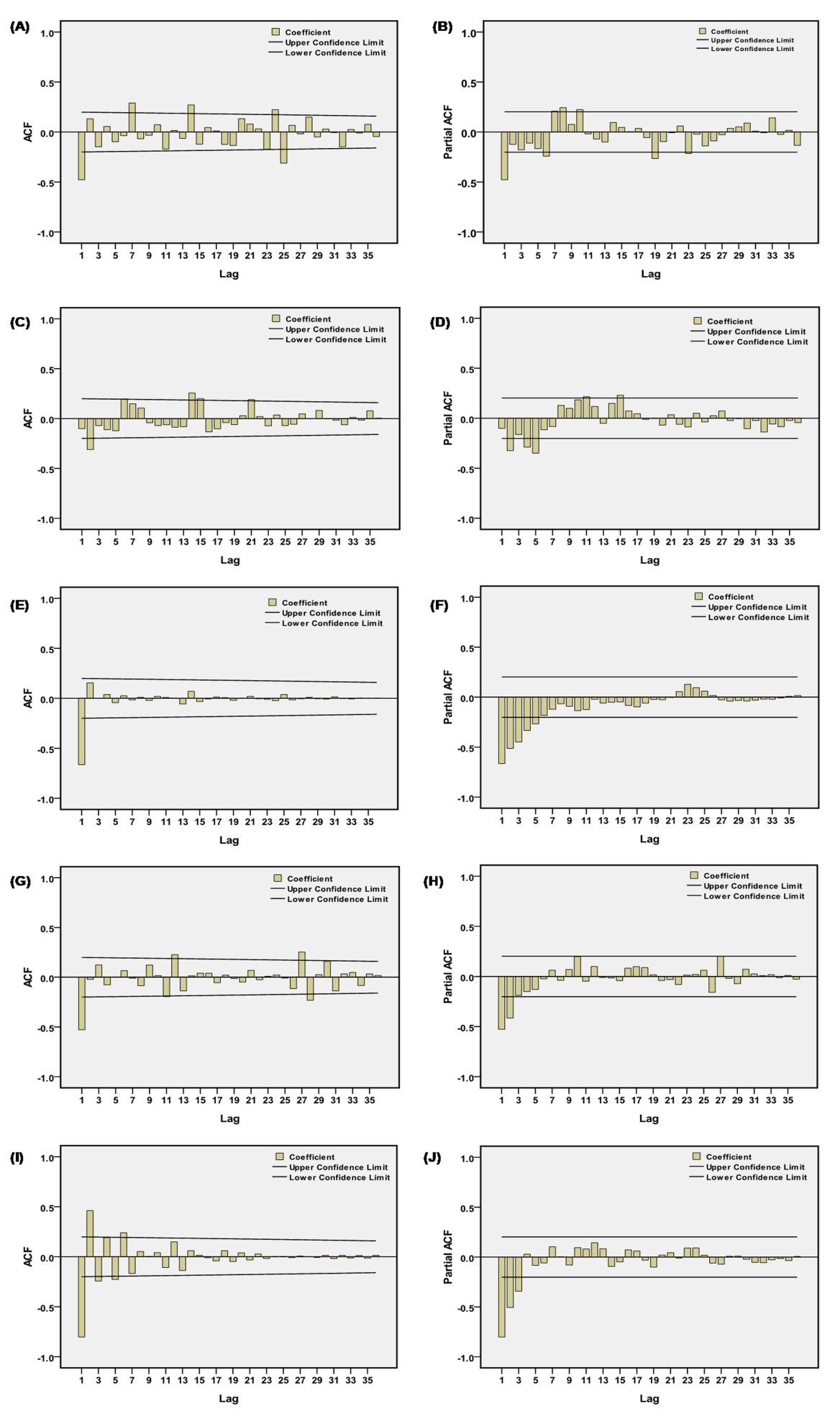


**Fig. S2.** Autocorrelation function (ACF) and partial ACF (PACF) plots showing the differenced prevalence series in the five regions. (A) ACF plot for the second-order differenced prevalence series globally; (B) PACF plot for the second-order differenced prevalence series globally; (C) ACF plot for the second-order differenced prevalence series in Brazil; (D) PACF plot for the second-order differenced prevalence series in Brazil; (E) ACF plot for the second-order differenced prevalence series in Peru; (F) PACF plot for the second-order differenced prevalence series in Peru; (G) ACF plot for the second-order differenced prevalence series in Canada; (H) PACF plot for the second-order differenced prevalence series in Canada; (I) ACF plot for the second-order differenced prevalence series in Chile; (J) PACF plot for the second-order differenced prevalence series in Chile. We could see from the differenced prevalence series correlogram that the autocorrelations and partial autocorrelations at various lags were touching the significance bounds, and thus the orders of autoregression (AR) and moving average (MA) could be crudely chosen with the help of these differenced prevalence series plots.


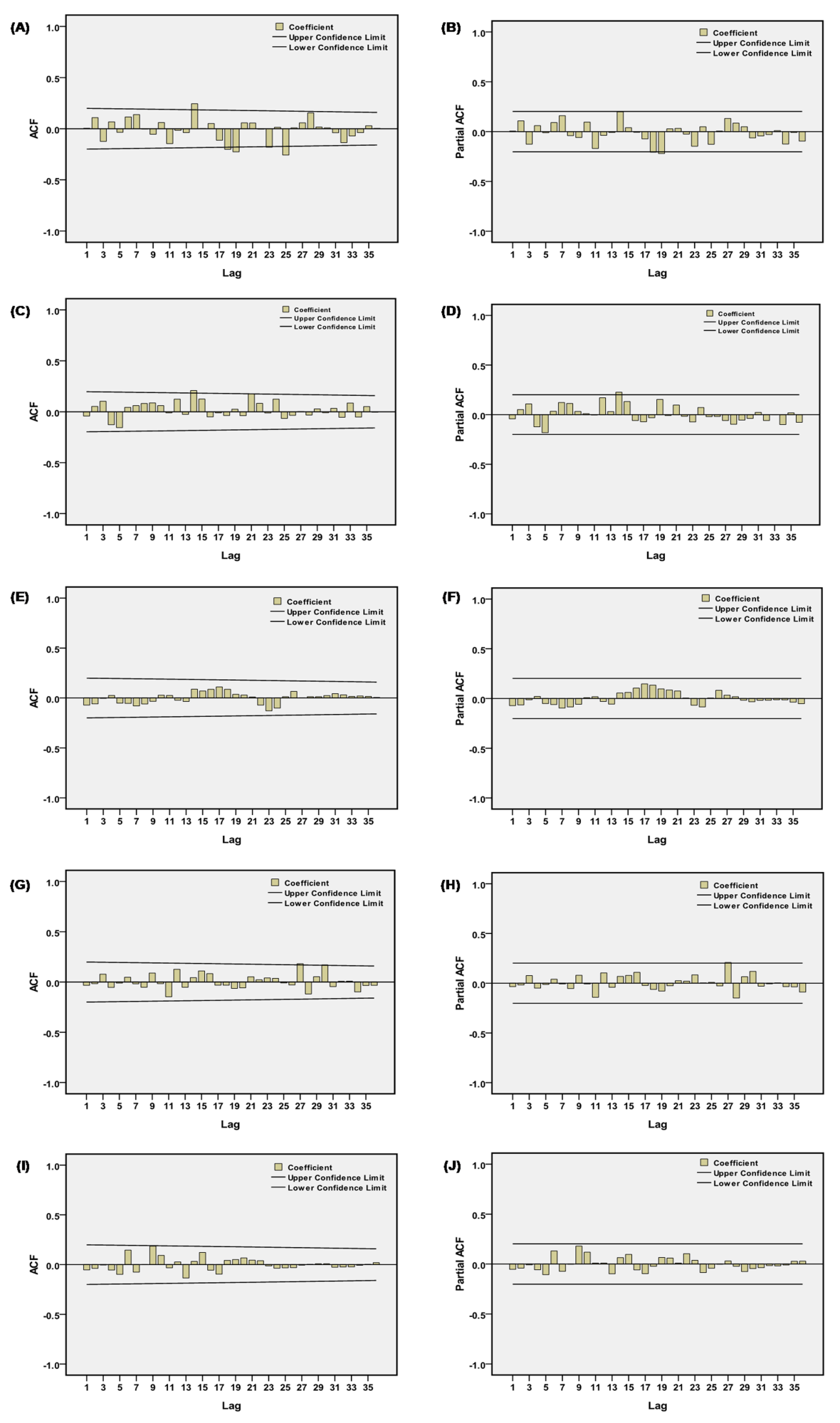


**Fig. S3.** Autocorrelation function (ACF) and partial ACF (PACF) plots of the residual series to estimate the epidemiological trends of the COVID-19 prevalence in the five regions. (A) ACF plot globally; (B) PACF plot globally; (C) ACF plot in Brazil; (D) PACF plot in Brazil; (E) ACF plot in Peru; (F) PACF plot in Peru; (G) ACF plot in Canada; (H) PACF plot in Canada; (I) ACF plot in Chile; (J) PACF plot in Chile. As shown in the above plots, almost all the correlation coefficients fell into the estimated 95% uncertainty levels, indicating the identified best ARIMA models are adequate for the prevalence data. Sometime, we may find some higher-order correlation coefficients outside of the 95% uncertainty levels, which is also reasonable because it readily occurs by chance.


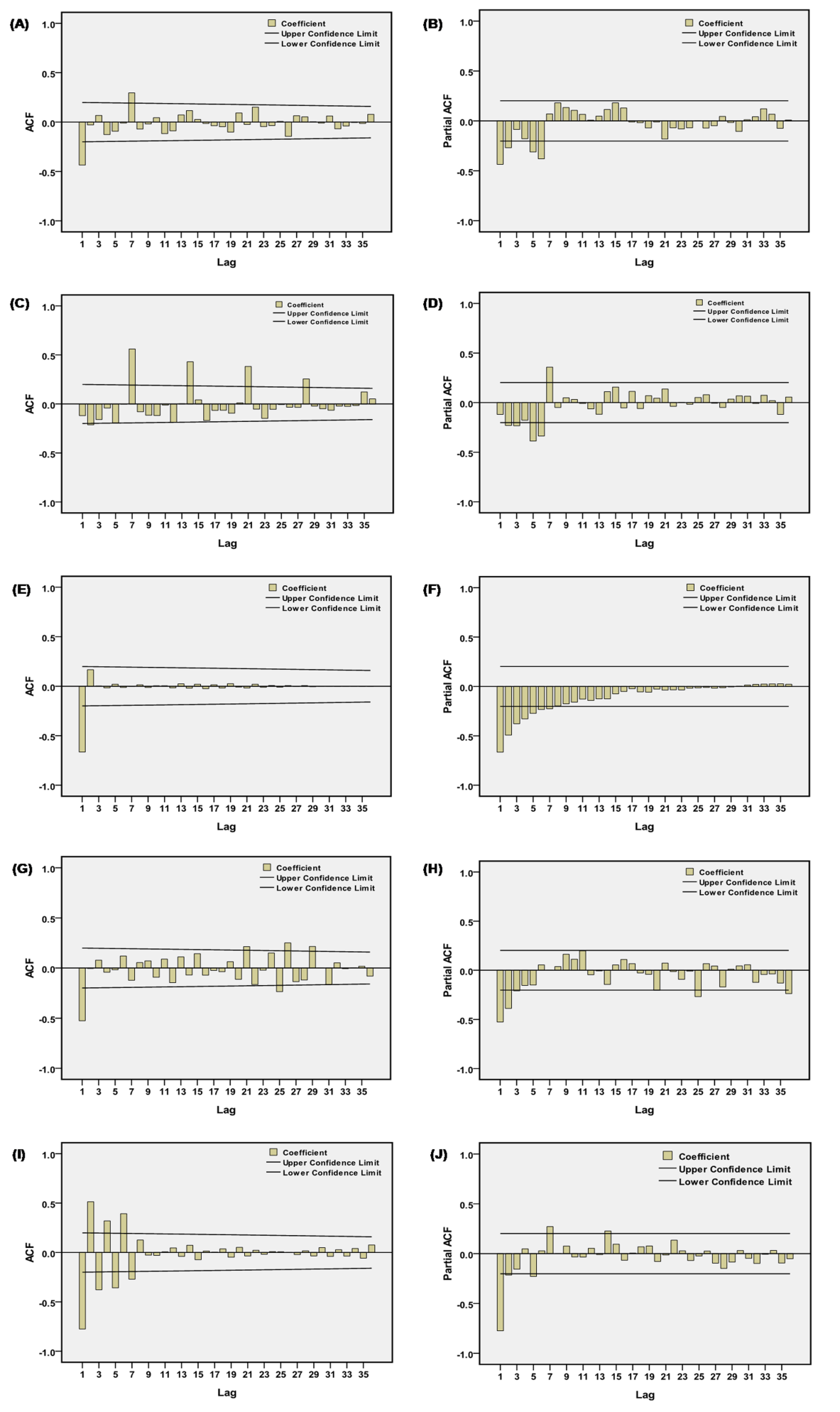


**Fig. S4.** Autocorrelation function (ACF) and partial ACF (PACF) plots showing the differenced mortality series in the five regions. (A) ACF plot for the second-order differenced mortality series globally; (B) PACF plot for the second-order differenced mortality series globally; (C) ACF plot for the second-order differenced mortality series in Brazil; (D) PACF plot for the second-order differenced mortality series in Brazil; (E) ACF plot for the second-order differenced mortality series in Peru; (F) PACF plot for the second-order differenced mortality series in Peru; (G) ACF plot for the second-order differenced mortality series in Canada; (H) PACF plot for the second-order differenced mortality series in Canada; (I) ACF plot for the second-order differenced mortality series in Chile; (J) PACF plot for the second-order differenced mortality series in Chile. We could see from the differenced mortality series correlogram that the autocorrelations and partial autocorrelations at various lags were touching the significance bounds, and thus the orders of autoregression (AR) and moving average (MA) could be roughly determined with the help of these differenced prevalence series plots.


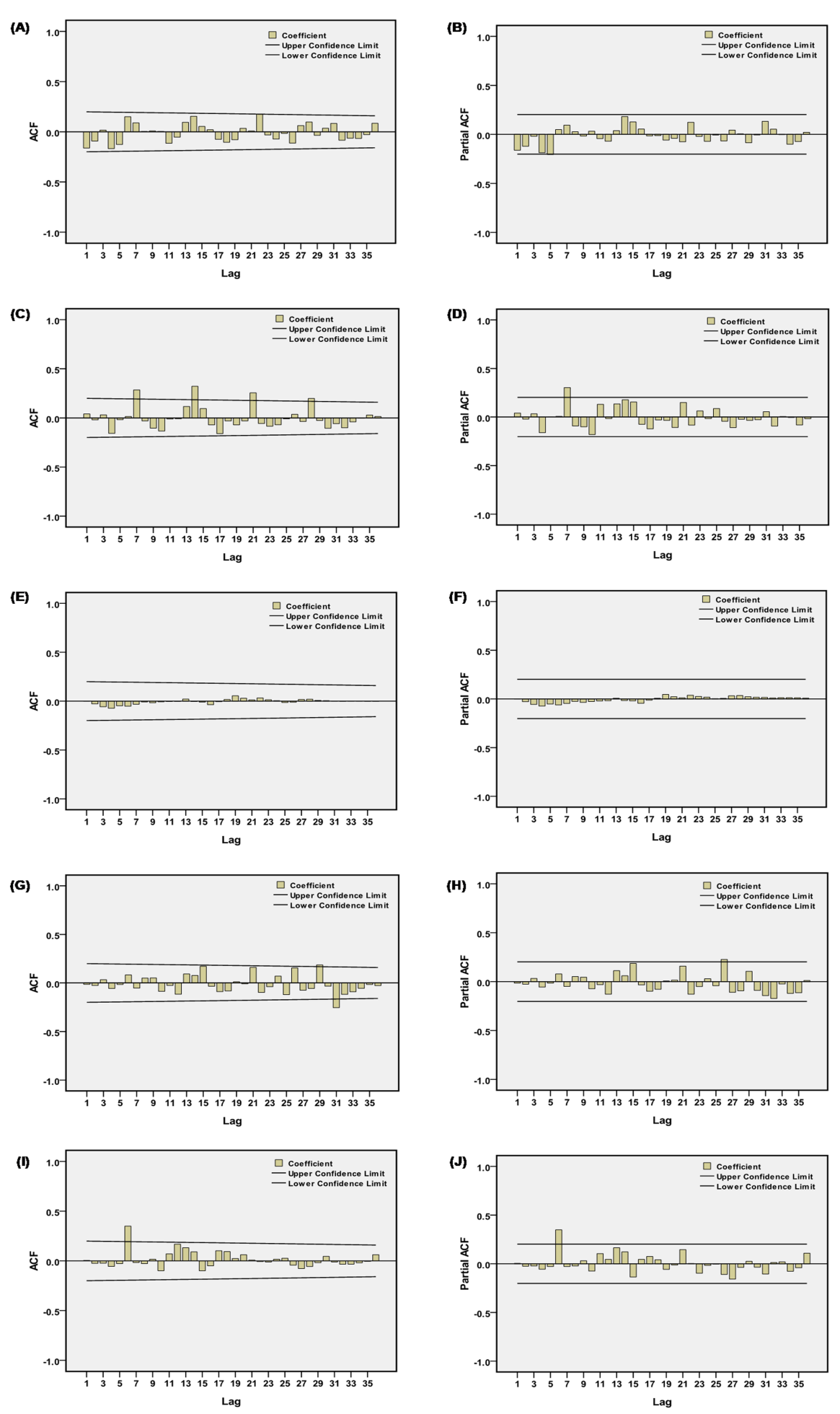


**Fig. S5.** Autocorrelation function (ACF) and partial ACF (PACF) plots of the residual series to estimate the epidemiological trends of the COVID-19 mortality in the five regions. (A) ACF plot globally; (B) PACF plot globally; (C) ACF plot in Brazil; (D) PACF plot in Brazil; (E) ACF plot in Peru; (F) PACF plot in Peru; (G) ACF plot in Canada; (H) PACF plot in Canada; (I) ACF plot in Chile; (J) PACF plot in Chile. As shown in the above plots, almost all the correlation coefficients fell into the estimated 95% uncertainty levels, indicating the identified best ARIMA models are adequate for the prevalence data. Sometimes, it can be found that the higher-order correlation coefficients lay outside the 95% uncertainty levels, this phenomenon is also reasonable because it readily occurs by chance.
